# Supplementary material for: N-Type Nanocomposite Films Combining SWCNTs, Bi2Te3 Nanoplates, and Cationic Surfactant for Pn-Junction Thermoelectric Generators with Self-Generated Temperature Gradient Under Uniform Sunlight Irradiation
Source: Sensors (Basel). 2024 Nov 1;24(21):7060. doi: 10.3390/s24217060 (PMC11548406; doi:10.3390/s24217060)
Supplement: Supplementary file 1 [file sensors-24-07060-s001.zip › sensors-3261400-supplementary.pdf]

N-type Nanocomposite Films Combining SWCNTs, Bi_2Te_3 Nanoplates, and Cationic Surfactant for Pn-junction Thermoelectric Generators with Self-Generated Temperature Gradient under Uniform Sunlight Irradiation

Koki Hoshino[1], Hisatoshi Yamamoto[1], Ryota Tamai[1], Takumi Nakajima[1], Shugo Miyake[2], and Masayuki Takashiri[1,*]

[1] Department of Materials Science, Tokai University, Hiratsuka, Kanagawa 259-1292, Japan

[2] Department of Mechanical Engineering, Setsunan University, Neyagawa, Osaka 572-8508, Japan

* Corresponding author. Email: takashiri@tokai.ac.jp (Masayuki Takashiri)

Supplementary Information

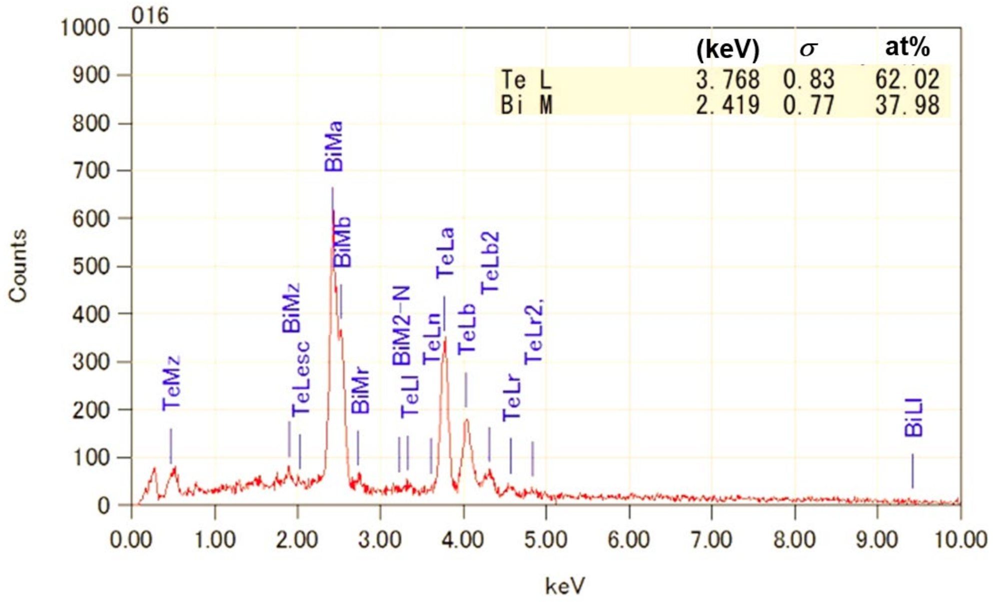

Figure S1. EDS spectrum and corresponding quantitative analysis of as-prepared Bi_2Te_3 nanoplates.

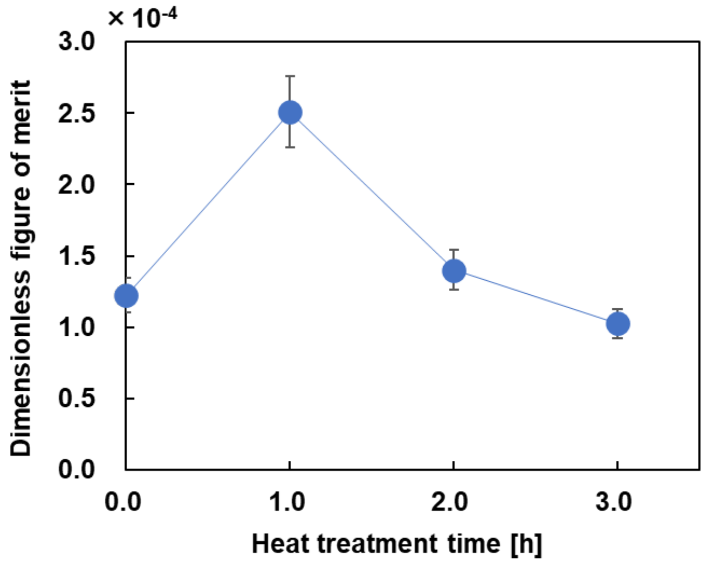

Figure S2. Dimensionless figure of merit of nanocomposite films as a function of heat treatment time.

The dimensionless figure of merit, ZT, is calculated using the following equations: $ZT = \sigma S^2 T/\kappa$, where σ, S, T, and κ are the electrical conductivity, Seebeck coefficient, absolute temperature, and thermal conductivity, respectively. The results show that the highest ZT of 2.5×10^{-4} was exhibited at a heat treatment time of 1 h.

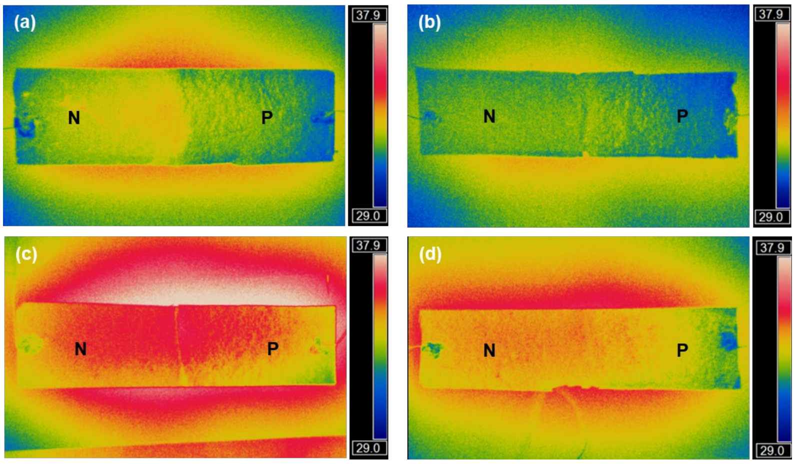

Figure S3. Thermographic images of pn-junction TEGs with (a) untreated film, and films heat-treated at (b) 1h, (c) 2h, and (d) 3h under uniform artificial sunlight irradiation for 800 s after light exposure.

The thermal distributions of the pn-junction TEGs were measured using a thermography camera (OPTRIS, OPTXI40LTF20CFKT090) for 800 s after sunlight irradiation. All pn-junction TEGs showed that the temperature gradient was created from the center of the film to both edges of the film.

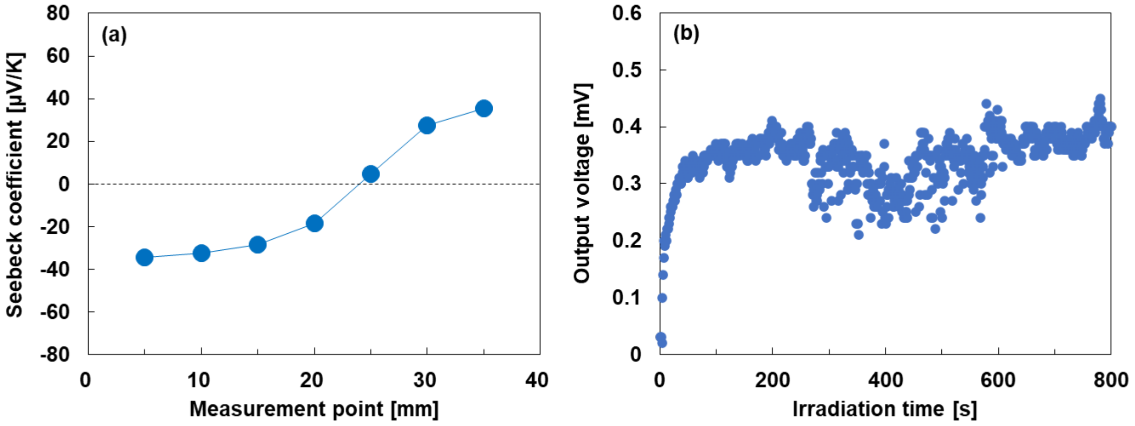

Figure S4. Performance of the pn-junction TEG without Bi_2Te_3 nanoplates heat-treated for 1 h. (a) Spatial distribution and (b) exposure time dependence of the output voltage.

To investigate the effect of Bi_2Te_3 nanoplates in the films, we fabricated the pn-junction TEG without Bi_2Te_3 nanoplates while the heat treatment time was maintained at 1 h. In Fig. S4(a), the spatial distribution of the Seebeck coefficient of the pn-junction TEG without Bi_2Te_3 nanoplates had a similar spatial distribution to that of the pn-junction TEG with Bi_2Te_3 nanoplates heat-treated for 1 h. In Fig. S4(b), the pn-junction TEG produced the output voltage exposed to artificial sunlight. The average output voltage from the exposure time of 200 to 800 s was 0.35 mV, which is lower than that of the pn-junction TEG with Bi_2Te_3 nanoplates heat-treated for 1 h.

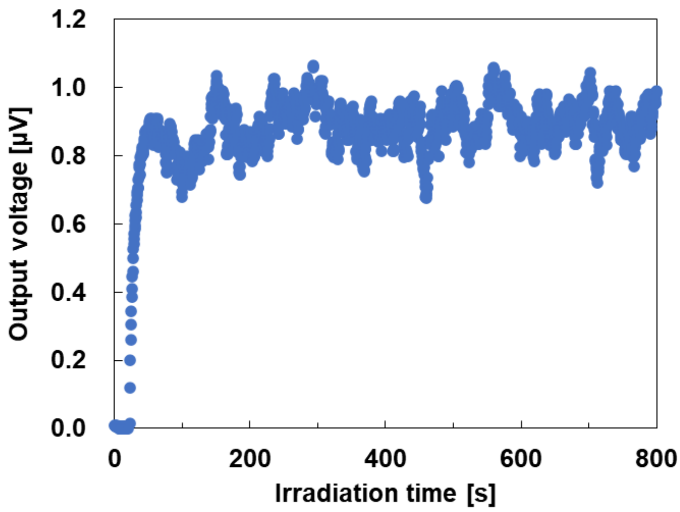

Figure S5. Exposure time dependence of the output voltage of the pn-junction TEG under uniform infrared radiation.

To investigate whether the pn-junction TEGs can act as infrared sensors, we measured the output voltage of the TEG under uniform infrared radiation. Here, we used a pn-junction TEG heat-treated for 1 h because it exhibited the highest output voltage when exposed to artificial sunlight. The experimental setup is based on measuring the pn-junction TEGs under artificial sunlight irradiation, as shown in Fig. 7(a). The performance of the TEGs was evaluated under uniform infrared irradiation using a graphite heater (Aladdin, AEH-G408N), where the wavelength was approximately 2–4 μm and the light intensity was approximately 100 W/m^2 when the distance between the infrared light source and the TEG was 200 mm. The output voltage of the TEGs was measured using a data logger (HIOKI, LR8432) for 800 s after infrared light exposure. Two copper wire electrodes were connected to both ends of

the film, and the opposite ends of those copper wire electrodes were connected to a data logger to measure the output voltage. The time dependence of output voltages generated in the pn-junction TEGs is shown in Fig. S5. The TEG showed stable voltage at approximately 200 s after uniform infrared irradiation. The average output voltage of the TEG in the flat region was 0.11 μV. Although the value is lower than that of the TEG when exposed to artificial sunlight due to the low irradiance of the infrared light, the pn-junction TEG can generate the output voltage under uniform infrared radiation. Therefore, pn-junction TEGs have the potential to act as infrared sensors.
